# Editorial: Insights in Psychological Therapies: 2021

**DOI:** 10.3389/fpsyt.2022.890889

**Published:** 2022-04-08

**Authors:** Takeshi Terao, Veena Kumari

**Affiliations:** ^1^Department of Neuropsychiatry, Faculty of Medicine, Oita University, Oita, Japan; ^2^Department of Life Sciences, College of Health, Medicine and Life Sciences, Brunel University London, Uxbridge, United Kingdom

**Keywords:** metacognitive therapy, narrative exposure therapy, dialectical behavior therapy, psychodrama, existential intervention

The year 2021 may be called a year of anxiety and stress because of the ongoing mental health toll of the Covid-19 pandemic, recession, change of workstyle, and the digital revolution. Therefore, the need for effective psychological therapies at present is much greater than before. On this Topic, we highlight six excellent papers as follows.

First, both De Dominicis et al. and Hansmeier et al. investigated the effects of metacognitive therapy. In contrast to wellknown cognitive behavior therapy, metacognitive therapy does not focus on the client's thought content, but on metacognition, which is the awareness or analysis of one's own thinking process about their thoughts. De Dominicis et al. applied metacognitive therapy to four individuals suffering from chronic work-related stress. Specifically, the therapist challenged clients' positive and negative metacognitive beliefs *via* Socratic dialogue and behavioral experiments and provided the client with a new set of responses, alternative to the cognitive-attentional syndrome (CAS) consisting of heightened self-focused attention, reduced efficiency of cognitive functioning, activation of self-beliefs and self-appraisal, attentional bias, and capacity limitations. The therapy effectiveness depends on whether CAS has been reduced so that meta-worry and meta-beliefs have changed permanently. Encouragingly, their findings showed significant improvements not only in general mental health, perceived stress, and blood pressure, but also in maladaptive coping strategies, avoidance behaviors, and depression symptoms, suggesting the usefulness of metacognitive therapy for work-related stress. As this was the first successful attempt to address chronic work-related stress with metacognitive therapy, we look forward to its confirmation with robust study designs in future studies.

Hansmeier et al. performed metacognitive therapy in 12 obsessive-compulsive disorder (OCD) patients and exposure with response prevention in 12 OCD patients and compared the effects of these two psychotherapies on OCD-specific metacognition. Verbal methods (e.g., Socratic questioning about evidence, reframing advantages), detached mindfulness, and behavioral experiments (e.g., ritual postponement) were applied during metacognitive therapy to change metacognition. Both treatments led to a significant change from pre- to post-treatment in all three OCD-specific metacognitions such as thought-action fusion beliefs, positive beliefs about rituals, and stop signals. Of note, metacognitive therapy showed a significantly stronger effect in reducing thought-action fusion beliefs than exposure with response prevention, although there was no difference between treatments in reducing beliefs about rituals or stop signals.

Hartmann et al. reported a study protocol of a therapist-guided internet-based cognitive-behavioral program for adolescents and young adults with body dysmorphic disorder to evaluate the efficacy and its superiority over supportive online therapy conditions. Fruitful results are expected in the near future.

Steuwe et al. randomized 60 female patients with borderline personality disorder (BPD) and comorbid post-traumatic stress disorder to either a 10-week residential narrative exposure therapy (NET) or dialectical behavior therapy-based treatment (DBT-bt). Although DBT has been shown to be effective in BPD, it was not found to be suffciently effective for PTSD symptomatology in patients with BPD and PTSD. On the other hand, NET aims to combine highly emotional trauma memories with the correct situational and temporal contextual information, promoting a coherent autobiographical memory associated with sensory, affective, and cognitive features of the experience. NET was specifically designed and proven to be effective for patients who have experienced multiple events and different types of traumatic experiences. As BPD patients with PTSD commonly experience many traumatic events, NET could be advantageous for these patients. Steuwe et al. hypothesized that NET will be superior to DBT-bt at 12-months follow-up with regard to reductions in PTSD symptom severity. Their hypotheses were partially confirmed by the study results because NET showed a significantly higher remission rate than DBT-bt and noteworthy PTSD remission was in all cases accompanied by BPD remission.

Lim et al. performed a scientometric review about psychodrama. Psychodrama is a form of psychotherapy that utilizes elements of theater, role-play, and group dynamics, which was formally conceptualized by Moreno at the turn of the 1920s in the aftermath of World War I. It was built upon the idea of an “action method” that allowed participants to act out their problems rather than merely talking about them in traditional “talk therapy.” Lim et al. performed document co-citation analysis (DCA) and found that psychodrama has had a rich, yet complex, history in research, suggesting that in order to enable a unifying view of psychodrama there is a strong need for more transparency in the implementation of psychodramatic programs that various studies and researchers take.

Finally, Terao and Satoh reviewed the literature on existential psychotherapies dealing with patients suffering from advanced cancer and/or terminal care. Their review found nine types of existential psychotherapies which were investigated using randomized controlled trials: meaning-centered group psychotherapy (MCGP), individual meaning-centered psychotherapy (IMCP), meaning-making intervention (MMi), meaning of life intervention, managing cancer and living meaningfully (CALM), hope intervention, cognitive and existential intervention, dignity therapy, and life-review interviews. All of these therapies deal with existential concerns, such as death, meaninglessness, isolation, and freedom. Particularly, MCGP, IMCP, MMi, meaning of life intervention, and CALM emphasize finding and/or making meaning in the individual's life. The effects on existential or spiritual wellbeing were confirmed in MCGP, IMCP, meaning of life intervention, and life-review intervention. Since the number of studies was very small, further studies are required to investigate the effects of existential psychotherapy on patients with advanced cancer and/or terminal care.

In this Topic, we can see metacognitive therapy, narrative exposure therapy, dialectical behavior therapy, psychodrama, and existential intervention, although some are new (or applied in a new context) and others are old. It is interesting that any therapy, whether it is new or old, has more or less existential factors and places emphasis on the individual patient's (client's) life. To conquer the stress and anxiety of clients and patients, there is a strong need for our clinicians and psychiatrists to support their lives individually and elaborately.

## Author Contributions

TT wrote this editorial. VK constructively revised it. Both approved of the final version.

## Conflict of Interest

The authors declare that the research was conducted in the absence of any commercial or financial relationships that could be construed as a potential conflict of interest.

## Publisher's Note

All claims expressed in this article are solely those of the authors and do not necessarily represent those of their affiliated organizations, or those of the publisher, the editors and the reviewers. Any product that may be evaluated in this article, or claim that may be made by its manufacturer, is not guaranteed or endorsed by the publisher.

